# Systolic Blood Pressure Trajectory and Outcomes in Acute Intracerebral Hemorrhage: Pooled Analysis of the 4 INTERACT and ATACH-II Clinical Trials

**DOI:** 10.1212/WNL.0000000000214671

**Published:** 2026-03-19

**Authors:** Xia Wang, Thanh G. Phan, Xinwen Ren, Henry H. Ma, Qiang Li, Menglu Ouyang, Candice Delcourt, Xiaoying Chen, Jiguang Wang, Thompson G. Robinson, Hisatomi Arima, Lu Ma, Xin Hu, Chao You, Leibo Liu, Paula Munoz Venturelli, Sheila Co Martins, Octavio M. Pontes-Neto, John Philip Chalmers, Lili Song, Adnan I. Qureshi, Craig Anderson

**Affiliations:** 1 The George Institute for Global Health, Faculty of Medicine, University of New South Wales, Sydney, Australia;; 2 Stroke & Aging Research Group, Monash University, Melbourne, Australia;; 3 Department of Neurology, Monash Health, Melbourne, Australia;; 4 Department of Clinical Medicine, Faculty of Medicine, Health and Human Sciences, Macquarie University, Sydney, Australia;; 5 Centre for Epidemiological Studies and Clinical Trials, Shanghai Institute of Hypertension, Ruijin Hospital, Shanghai Jiaotong University School of Medicine, China;; 6 Department of Cardiovascular Sciences and NIHR Biomedical Research Unit in Cardiovascular Disease, University of Leicester, United Kingdom;; 7 Department of Cardiovascular Sciences, University of Leicester, United Kingdom;; 8 Department of Preventive Medicine and Public Health, Faculty of Medicine, Fukuoka University, Japan;; 9 Department of Neurosurgery, West China Hospital, Sichuan University, Chengdu, China;; 10 Centre for Big Data Research in Health, Faculty of Medicine, University of New South Wales, Sydney, Australia;; 11 Clinica Alemana de Santiago, Facultad de Medicina, Clinica Alemana Universidad del Desarrollo, Santiago, Chile;; 12 Centro de Estudios Clínicos, Instituto de Ciencias e Innovación en Medicina, Facultad de Medicina, Clínica Alemana Universidad del Desarrollo, Santiago, Chile;; 13 Neurology Department, Hospital Moinhos de Vento, Porto Alegre, Brazil;; 14 Department of Neurology, Ribeirão Preto Medical School, University of São Paulo, Brazil;; 15 Institute for Science and Technology for Brain-inspired Intelligence, Fudan University, Shanghai, China; and; 16 Neurology Department, Royal Prince Alfred Hospital, Sydney, Australia.

## Abstract

**Background and Objectives:**

While moderate and rapid systolic blood pressure (SBP) lowering was associated with better functional outcomes after intracerebral hemorrhage (ICH), large reductions in SBP within 1 hour, for example, reductions from >200 to <140 mm Hg, diminished or even reversed these beneficial effects. We aimed to define the optimal trajectory of SBP control in relation to favorable functional outcomes after acute ICH.

**Methods:**

We conducted a pooled analysis of individual patient-level data from all 4 Intensive Blood Pressure Reduction in Acute Cerebral Hemorrhage Trial (INTERACT) and second Antihypertensive Treatment of Acute Cerebral Hemorrhage (ATACH-II) trials, which were international, randomized, open-label, blinded, end point–assessed, controlled trials that determined the effectiveness of early intensive SBP control in acute ICH. Latent class analysis grouped SBP trajectories over the first 24 hours (9 measurements) into defined clusters. The primary outcome was functional recovery at 90 days after randomization, defined as modified Rankin Scale (mRS) scores of 3–6. Logistic regression models with adjustment for baseline covariates and trial were used to determine associations between SBP cluster trajectories and outcomes in INTERACT, with validation in ATACH-II.

**Results:**

A total of 11,269 patients (INTERACT n = 10,269; ATACH-II n = 1,000; mean age 62.4 years; female 36.4%) with at least 1 postrandomization SBP reading were included. Six SBP trajectories were identified: low, moderate-to-low, moderate, high, high-to-moderate, and high-to-low. Compared with the low SBP group, associations with poor functional outcome (mRS scores 3–6) increased progressively across other groups in INTERACT (*p* = 0.04 for trend). Adjusted odds ratios (95% CI) for groups 2 to 6 were 1.16 (0.98–1.37), 1.44 (1.18–1.75), 1.46 (1.15–1.87), 1.90 (1.32–2.73), and 1.28 (1.02–1.60), respectively. A similar albeit nonsignificant trend was observed in ATACH-II due to limited power.

**Discussion:**

Distinct SBP trajectories over 24 hours defined prognosis after ICH, with a severe hypertensive group having the highest odds of death or disability, regardless of the BP-lowering strategy used. These findings highlight the importance of well-controlled but tailored SBP management strategies after ICH.

**Classification of Evidence:**

This study provides Class III evidence that distinct SBP trajectories over 24 hours are associated with prognosis after acute ICH, with a severe hypertensive group having the highest odds of poor functional outcome, regardless of the BP-lowering strategy used.

**Trial Registration Information:**

(INTERACT1 NCT00226096; INTERACT2 NCT00716079; INTERACT3 NCT03209258; INTERACT4 NCT03790800; ATACH-2 NCT01176565).

## Introduction

A pooled analysis of individual patient-level data (IPD) from the second Intensive Blood Pressure Reduction in Acute Cerebral Hemorrhage Trial (INTERACT2) and the second Antihypertensive Treatment of Acute Cerebral Hemorrhage (ATACH-II) trial indicates that careful titration and maintenance of smooth systolic blood pressure (SBP) in the first 24 hours after the onset of acute spontaneous intracerebral hemorrhage (ICH) provides beneficial effects.^1,2^ Each 10-mm Hg reduction in mean SBP is associated with a 10% improvement in the odds of good functional recovery, down to SBP levels as low as 120–130 mm Hg. However, while moderate and rapid SBP lowering was associated with a better functional outcome after ICH, large reductions in SBP within 1 hour, for example, reductions from >200 to <140 mm Hg, diminished or even reversed these beneficial effects.^3^ No study has collectively examined and quantified differences in the magnitude of associations between various patterns or trajectories of SBP reduction and clinical outcomes. Moreover, it is unclear whether there may be differential effects according to different approaches to SBP control: maintaining a high SBP >180 mm Hg in the first hour and >160 mm Hg over the first 24 hours; implementing moderate reduction, for example, lowering SBP from >200 to <200 mm Hg in the first hour and then maintaining at the level of >150 mm Hg over the first 24 hours; significant lowering, for example, from 200 to 160 mm Hg in the first hour and maintaining >130 mm Hg over the first 24 hours.^4^ In this study, we provide results of our use of a trajectory modeling approach, defined before statistical analysis, to explore the associations between SBP trajectories and clinical outcome using pooled IPD from all 5 clinical trials: INTERACT^5-8^ and ATACH-II.^9^ The primary research question addressed in this study was to determine which SBP trajectories are associated with improved clinical outcomes.

## Methods

### Design

Both INTERACT1^5^ and INTERACT2^6^ were international, multicenter, open-label, blinded, end point–assessed, randomized controlled trials including patients with acute ICH (<6 hours of onset) and elevated SBP (150–220 mm Hg) who were allocated to receive intensive (target SBP <140 mm Hg within 1 hour) or guideline-recommended (target SBP <180 mm Hg) BP-lowering treatment. The third INTEnsive care bundle with blood pressure Reduction in Acute Cerebral haemorrhage Trial (INTERACT3)^7^ was an international, multicenter, prospective, stepped-wedge, cluster-randomized, blinded outcome–assessed, controlled trial in which clusters of patients were prospectively followed up to establish their outcome as hospitals were randomly allocated to switch from control “usual care” to intervention “implementation of a care bundle” at different time points. Hospitals were eligible if they had no or inconsistent, disease-specific protocols for ICH and were willing to implement the care bundle that included early intensive BP lowering (target SBP <140 mm Hg), glucose control (target 6.1–7.8 mmol/L in those without diabetes mellitus and 7.8–10.0 mmol/L in those with diabetes), antipyrexia treatment (target body temperature ≤37.5°C), and rapid reversal of warfarin-related anticoagulation (target international normalized ratio <1.5). In the fourth INTEnsive ambulance-delivered BP Reduction in hyper-ACute stroke Trial (INTERACT4),^8^ patients had their BP monitored every 5 minutes for the first 30 minutes and then every 15 minutes until 60 minutes while in the ambulance during transport. After arriving at the hospital, BP was measured at 1, 2, 3, 4, 5, 6, 12, 18, and 24 hours. We assumed a 10-minute transfer time from the ambulance to the hospital emergency center and combined the ambulance and hospital measurements from the time of randomization. For the purposes of these analyses, the shared time points were time of randomization, every 15 minutes in 1 hour (when the largest drop in SBP was assumed to have occurred), and then at 6, 12, 18, and 24 hours. In ATACH-II,^9^ patients were randomized to intensive (target SBP 110–139 mm Hg) or guideline-recommended (140–179 mm Hg) BP management with intravenous nicardipine within 4.5 hours of the onset of symptoms, with the aim of achieving target levels within 2 hours.

### Procedures

Key demographic and clinical characteristics were recorded at the time of enrollment, including neurologic severity measured using the NIH Stroke Scale (NIHSS). Patients were followed up to 90 days after randomization by trained staff masked to treatment allocation. Analyses were conducted in a modified intention-to-treat population, comprising patients with sufficient data on SBP and the primary outcome of functional recovery defined by the distribution of scores on the modified Rankin Scale (mRS, range from 0 [no symptoms] to 6 [death]), at 90 days after randomization. Secondary outcomes were dependency (mRS scores 3–6) and death at 90 days. Safety outcomes included neurologic deterioration, defined as an increase from baseline of ≥4 points on the NIHSS or a decrease from baseline of ≥2 points on the Glasgow Coma Scale over 24 hours, and any fatal or nonfatal cardiac or renal serious adverse event, according to standard definitions, within 90 days.

### Statistical Analysis

Latent class analysis (LCA) was used to differentiate patterns (“trajectories”) of SBP over the first 24 hours after randomization. Using maximum likelihood estimation, the LCA computed the probability of each individual being assigned to a class to which they had the highest (posterior) probability of belonging.^10^ A final class was chosen to provide an optimal measure for meaningful interpretability and parsimony according to Akaike Information Classification and Bayesian Information Classification indices.^10^ Differences between trajectory classes were examined by χ^2^ and Kruskal-Wallis tests for categorical and continuous variables, respectively. LCA was performed using the gsem package in STATA version 9.2 (Stata Corporation, College Station, TX).

Because our initial evaluation of the mRS scores using an ordinal scale showed that the proportional odds assumption was violated, we reverted to using logistic regression for analyses of poor functional outcome (mRS scores 3–6). The regression models examined the association between different SBP trajectories and outcomes using significant covariates identified from univariate analysis, along with variables of trial and randomization included by default in the model. We independently examined associations in the INTERACT and ATACH-II trials to determine whether the magnitude and direction of associations were consistent across studies. Results are presented as odds ratios (ORs) with 95% CIs, and a 2-sided *p* < 0.05 was considered statistically significant. Patients with missing data on any of the aforementioned variables were excluded from multivariable analysis. SAS version 9.3 (SAS Institute, Cary, NC) was used for analyses.

### Standard Protocol Approvals, Registrations, and Patient Consents

The studies were approved by ethics committees at each site, and written informed consent was obtained from each patient or, where appropriate, an approved surrogate. All trials have been registered at the ClinicalTrials.gov: INTERACT1 NCT00226096; INTERACT2 NCT00716079; INTERACT3 NCT03209258; INTERACT4 NCT03790800; ATACH-2 NCT01176565.

### Data Availability

Deidentified IPD of the INTERACT studies used in these analyses may be shared through a formal request with a protocol from any qualified investigator to the Research Office of The George Institute for Global Health, Australia. Deidentified IPD from ATACH-II are available indefinitely at the National Institute of Neurological Disorders and Stroke data archive (ninds.nih.gov/); to gain access, requesters need to sign a data-access agreement.

## Results

### Patient Characteristics by 6 Patterns of SBP Trajectory

**Figure 1:**
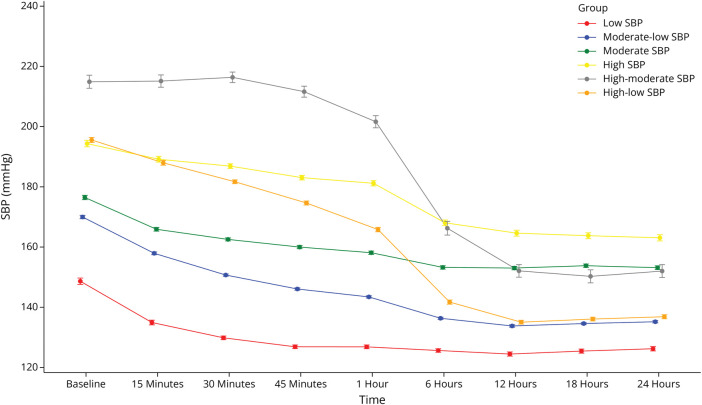
SBP Trajectories Over the First 24 Hours After Acute Intracerebral Hemorrhage SBP denotes systolic blood pressure. Time denotes time from onset of symptoms to randomization.

Included and excluded patient characteristics are given in eTable 1. Figure 1 illustrates temporal changes in SBP over 24 hours categorized into 6 trajectory groups: (1) “low,” (2) “moderate-to-low,” (3) “moderate,” (4) “high,” (5) “high-to-moderate,” and (6) “high-to-low” SBP. The mean SBP for each trajectory group is given in eTable 2. Group 1 (low SBP, n = 1,614) had a mean SBP of 149 mm Hg (95% CI 147.6–149.7) at baseline, decreasing to a range of 120–130 mm Hg over 24 hours. Group 2 (moderate-to-low SBP, n = 4,651) had a mean SBP of 170 mm Hg (169.4–170.5) at baseline, dropping to 143 mm Hg (143.1–143.8) at 1 hour and stabilizing in the range of 130–140 mm Hg throughout 24 hours. Group 3 (moderate SBP, n = 2,310) had a baseline mean SBP of 177 mm Hg (175.7–177.2), ranging from 150 to 160 mm Hg over 24 hours. Group 4 (high SBP, n = 1,165) had a mean SBP of 194 mm Hg (193.3–195.4) at baseline, 181 mm Hg (180.3–182.0) at 1 hour, and a range of 160–170 mm Hg over 24 hours. Group 5 (high-to-moderate SBP, n = 437) had a mean SBP of 215 mm Hg (212.7–217.1) at baseline, 202 mm Hg (199.6–203.7) at 1 hour, and a range of 150–170 mm Hg over 24 hours. Group 6 (high-to-low SBP, n = 2,128) had a baseline mean SBP of 196 mm Hg (194.7–196.4), dropping to 166 mm Hg (165.1–166.5) at 1 hour and stabilizing in the range of 130–140 mm Hg over 24 hours.

Overall, 12,305 patients with at least 1 SBP reading in the first 24 hours after randomization (INTERACT, n = 11,305, eTable 3; ATACH-II, n = 1,000, eTable 4) were included in these analyses. Table 1 summarizes the baseline characteristics of patients categorized into the 6 SBP trajectory groups. Overall, their mean age was 62.6 (SD 12.7) years and 4,444 (36.1%) were female, with a median baseline neurologic impairment score on the NIHSS of 12 (interquartile range [IQR] 7–19). The mean SBP at randomization was 176.7 mm Hg (SD 25.3), with the median time from symptom onset to receipt of various BP-lowering strategies being 2.9 hours (IQR 1.9–4.0). The baseline median hematoma volume on the diagnostic CT brain scan was 13.0 mL (IQR 6.5–25.0).

**Table 1:** Baseline Characteristics of Patients by SBP Trajectories

Characteristics	SBP group							*p* Value
	Low (N = 1,614)	Moderate-low (N = 4,651)	Moderate (N = 2,310)	High (N = 1,165)	High-moderate (N = 437)	High-low (N = 2,128)	All (N = 12,305)	
Age, y	63 (12.9)	63 (12.4)	63 (12.8)	62 (13.3)	61 (13.4)	63 (12.7)	63 (12.7)	0.004
Sex, female	618/1,614 (38.3)	1,696/4,651 (36.5)	830/2,310 (35.9)	400/1,165 (34.3)	147/437 (33.6)	753/2,128 (35.4)	4,444/12,305 (36.1)	0.23
Region								<0.001
China	1,358/1,614 (84.1)	3,923/4,651 (84.3)	1,912/2,310 (82.8)	976/1,165 (83.8)	344/437 (78.7)	1,837/2,128 (86.3)	10,350/12,305 (84.1)	
India	157/1,614 (9.7)	463/4,651 (10.0)	309/2,310 (13.4)	113/1,165 (9.7)	25/437 (5.7)	162/2,128 (7.6)	1,229/12,305 (10.0)	
Latin America	18/1,614 (1.1)	54/4,651 (1.2)	26/2,310 (1.1)	28/1,165 (2.4)	20/437 (4.6)	26/2,128 (1.2)	172/12,305 (1.4)	
Other	81/1,614 (5.0)	211/4,651 (4.5)	63/2,310 (2.7)	48/1,165 (4.1)	48/437 (11.0)	103/2,128 (4.8)	554/12,305 (4.5)	
Randomization^a^								
Guideline-recommended	681/1,614 (42.2)	2,014/4,651 (43.3)	1,551/2,310 (67.1)	831/1,165 (71.3)	300/437 (68.6)	1,085/2,128 (51.0)	6,462/12,305 (52.5)	<0.001
Intensive	933/1,614 (57.8)	2,637/4,651 (56.7)	759/2,310 (32.9)	334/1,165 (28.7)	137/437 (31.4)	1,043/2,128 (49.0)	5,843/12,305 (47.5)	
Time to randomization	3.0 (2.0–4.0)	2.9 (1.8–4.0)	3.1 (2.0–4.2)	3.0 (2.0–4.1)	2.4 (1.3–3.7)	2.5 (1.5–3.9)	2.9 (1.9–4.0)	<0.001
Baseline SBP	148.7 (21.5)	170.0 (19.1)	176.5 (17.7)	194.3 (18.4)	214.9 (23.1)	195.5 (19.8)	176.7 (25.3)	<0.001
Baseline NIHSS score	12 (6–20)	12 (6–18)	12 (7–18)	13 (8–20)	15 (9–23)	13 (8–21)	12 (7–19)	<0.001
Baseline GCS score	13 (10–15)	13 (10–15)	14 (11–15)	13 (10–15)	12 (9–15)	13 (9–15)	13 (10–15)	<0.001
ICH volume, mL	14.0 (7.0–25.0)	12.0 (6.2–24.9)	12.3 (6.1–23.3)	13.8 (6.7–26.0)	15.0 (8.5–33.0)	13.8 (6.3–26.1)	13.0 (6.5–25.0)	<0.001
ICH location								0.001
Lobar	183/1,539 (11.9)	420/4,293 (9.8)	205/2,039 (10.1)	105/1,024 (10.3)	50/404 (12.4)	178/1,984 (9.0)	1,141/11,283 (10.1)	
Basal ganglia/deep	1,245/1,539 (80.9)	3,546/4,293 (82.6)	1,666/2,039 (81.7)	822/1,024 (80.3)	322/404 (79.7)	1,597/1,984 (80.5)	9,198/11,283 (81.5)	
Infratentorial	111/1,539 (7.2)	327/4,293 (7.6)	168/2,039 (8.2)	97/1,024 (9.5)	32/404 (7.9)	209/1,984 (10.5)	944/11,283 (8.4)	
IVH	426/1,600 (26.6)	1,280/4,553 (28.1)	660/2,219 (29.7)	379/1,121 (33.8)	179/431 (41.5)	653/2,081 (31.4)	3,577/12,005 (29.8)	<0.001
Hypertension	997/1,611 (61.9)	3,281/4,642 (70.7)	1,723/2,303 (74.8)	903/1,163 (77.6)	338/437 (77.3)	1,559/2,118 (73.6)	8,801/12,274 (71.7)	<0.001
Diabetes mellitus	169/1,613 (10.5)	495/4,642 (10.7)	275/2,306 (11.9)	153/1,164 (13.1)	52/436 (11.9)	226/2,122 (10.7)	1,370/12,283 (11.2)	0.12
Stroke^b^	250/1,536 (16.3)	668/4,316 (15.5)	385/2,139 (18.0)	175/1,079 (16.2)	57/360 (15.8)	261/1,828 (14.3)	1,796/11,258 (16.0)	0.04
Ischemic heart disease	140/1,613 (8.7)	352/4,649 (7.6)	178/2,310 (7.7)	83/1,165 (7.1)	21/437 (4.8)	116/2,124 (5.5)	890/12,298 (7.2)	0.001
Antihypertensive	618/1,612 (38.3)	1,914/4,647 (41.2)	1,059/2,308 (45.9)	513/1,164 (44.1)	185/437 (42.3)	861/2,122 (40.6)	5,150/12,290 (41.9)	<0.001
Antiplatelet	103/1,481 (7.0)	245/4,273 (5.7)	155/2,038 (7.6)	84/1,117 (7.5)	29/423 (6.9)	98/1,969 (5.0)	714/11,301 (6.3)	0.003
Anticoagulation	50/1,480 (3.4)	91/4,270 (2.1)	45/2,038 (2.2)	17/1,117 (1.5)	6/423 (1.4)	37/1,967 (1.9)	246/11,295 (2.2)	0.014

Abbreviations: GCS = Glasgow Coma Scale; ICH = intracerebral hemorrhage; IVH = intraventricular hemorrhage; NIHSS = NIH Stroke Scale; SBP = systolic blood pressure.

Data are n/N (%), mean (SD), or median (interquartile range); *p* values from χ^2^ or Kruskal-Wallis tests.

a Intensive (target SBP <140 mm Hg within 1 hour) or guideline-recommended (target SBP <180 mm Hg).

b Intracerebral hemorrhagic stroke and ischemic stroke.

Compared with other groups, the low SBP group was more likely to be assigned to intensive BP-lowering treatment and to have had a lower baseline SBP, but less likely to have had a history of hypertension or be taking antihypertensive medications.

### Association of SBP Trajectory With 90-Day Outcomes

**Figure 2:**
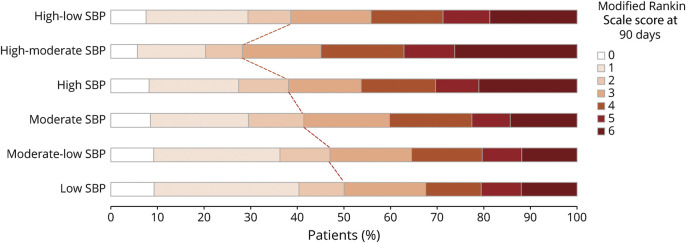
Distributions of Modified Rankin Scale Scores by SBP Trajectories Scores on the modified Rankin Scale range from 0 to 6, with 0 indicating no symptoms, 1 symptoms without clinically significant disability, 2 slight disability, 3 moderate disability, 4 moderate-to-severe disability, 5 severe disability, and 6 death. SBP = systolic blood pressure.

The distributions of mRS scores by SBP trajectories are shown in Figure 2. Compared with group 1 (low SBP), the association between SBP trajectory and 90-day mRS scores 3–6 progressively increased across groups 2–6 (*p* = 0.004 for trend) in the INTERACT trials. As given in Table 2, the adjusted ORs and 95% CIs were 1.16 (0.99–1.36), 1.48 (1.23–1.79), 1.47 (1.15–1.86), 1.88 (1.33–2.65), and 1.37 (1.11–1.69) for groups 2 through 6, respectively. We note that the number of cardiac and renal serious adverse events was low, especially in ATACH-II, leading to very wide CIs and limiting the precision of these estimates.

**Table 2:** Associations of SBP Trajectories and Outcomes in the INTERACT and ATACH-II Trials

Outcome	Group	n/N (%)	INTERACT studies				ATACH-II			
			Adjusted OR^a^ (95% CI)	*p* Value	*p* Trend	*p* for global test	Adjusted OR^a^ (95% CI)	*p* Value	*p* Trend	*p* for global test
mRS scores 3–6	Low SBP	666/1,351 (49.3)	1.0		0.004	0.001	1.0		0.14	0.10
	Moderate-low SBP	2,078/3,928 (52.9)	1.16 (0.99–1.36)	0.06			1.02 (0.60–1.73)	0.95		
	Moderate SBP	1,134/1,928 (58.8)	1.48 (1.23–1.79)	<0.001			1.33 (0.68–2.59)	0.40		
	High SBP	652/1,050 (62.1)	1.47 (1.15–1.86)	0.002			1.61 (0.58–4.46)	0.36		
	High-moderate SBP	279/390 (71.5)	1.88 (1.33–2.65)	<0.001			8.26 (1.72–39.65)	0.008		
	High-low SBP	1,121/1,812 (61.9)	1.37 (1.11–1.69)	0.03			1.22 (0.60–2.47)	0.59		
Death	Low SBP	167/1,393 (12.0)	1.0		0.52	0.009	1.0		0.99	0.93
	Moderate-low SBP	486/4,002 (12.1)	1.06 (0.86–1.31)	0.57			0.64 (0.23–1.74)	0.38		
	Moderate SBP	296/1,953 (15.2)	1.18 (0.93–1.51)	0.18			0.86 (0.25–2.97)	0.81		
	High SBP	228/1,070 (21.3)	1.49 (1.11–1.98)	0.01			0.87 (0.15–5.23)	0.88		
	High-moderate SBP	104/398 (26.1)	0.97 (0.65–1.44)	0.86			1.33 (0.11–15.73)	0.82		
	High-low SBP	356/1,858 (19.2)	1.11 (0.85–1.44)	0.44			0.74 (0.21–2.6)	0.63		
Cardiac SAE	Low SBP	9/1,481 (0.6)	1.0		0.82	0.32			0.30	0.82
	Moderate-low SBP	24/4,273 (0.6)	0.38 (0.15–0.94)	0.04			1.95 (0.37–10.23)	0.43		
	Moderate SBP	22/2,038 (1.1)	0.65 (0.26–1.66)	0.37			2.45 (0.36–16.6)	0.36		
	High SBP	13/1,117 (1.2)	0.61 (0.21–1.79)	0.37			4.04 (0.35–46.36)	0.26		
	High-moderate SBP	4/423 (1.0)	0.40 (0.08–1.94)	0.26			7.54 (0.38–150.73)	0.19		
	High-low SBP	15/1,973 (0.8)	0.50 (0.17–1.44)	0.20			3.03 (0.44–21.00)	0.26		
Renal SAE	Low SBP	1/1,481 (0.1)	1.0		0.58	0.45	1.0		0.91	0.28
	Moderate-low SBP	8/4,273 (0.2)	1.79 (0.22–14.60)	0.59			0.47 (0.06–3.47)	0.46		
	Moderate SBP	7/2,083 (0.3)	2.17 (0.25–18.60)	0.48			—			
	High SBP	11/1,117 (1.0)	4.36 (0.50–38.23)	0.18			—			
	High-moderate SBP	6/423 (1.4)	5.43 (0.52–57.03)	0.16			3.87 (0.10–151.98)	0.47		
	High-low SBP	7/1,973 (0.4)	1.96 (0.21–18.11)	0.55			0.66 (0.04–10.65)	0.77		

Abbreviations: ATACH-II = second Antihypertensive Treatment of Acute Cerebral Hemorrhage; INTERACT = Intensive Blood Pressure Reduction in Acute Cerebral Hemorrhage Trial; mRS = modified Rankin Scale; NIHSS = NIH Stroke Scale; OR = odds ratio; SAE = serious adverse event; SBP = systolic blood pressure.

a Adjusted for trials, randomization, age sex, region, time from onset to randomization, baseline SBP, NIHSS score, baseline hematoma volume, hematoma location, history of hypertension, history of ischemic heart disease, use of antihypertensive medications, and intraventricular hemorrhage.

Compared with group 1, the association between the SBP trajectory and 90-day death was not significantly different across groups 2 to 6 (*p* = 0.519 for trend) in the INTERACT trials. As given in Table 2, the adjusted ORs and 95% CIs were 1.06 (0.86–1.31), 1.18 (0.93–1.51), 1.49 (1.11–1.98), 0.97 (0.65–1.44), and 1.11 (0.85–1.44) for groups 2 through 6, respectively. A similar trend was also observed in ATACH-II. Associations between SBP trajectories and cardiac/renal serious adverse events were similarly not significantly different across the 6 SBP trajectories in both INTERACT and ATACH-II trials.

### Classification of Evidence

This study provides Class III evidence that distinct SBP trajectories over 24 hours are associated with prognosis after acute ICH, with a severe hypertensive group having the highest odds of poor functional outcome, regardless of the BP-lowering strategy used.

## Discussion

These post hoc analyses of the INTERACT and ATACH-II trials identified 6 distinct SBP trajectory groups within the first 24 hours of postrandomization treatment with different intensities of BP-lowering treatment in acute ICH. Among these, the low SBP group had the lowest risk of death or disability at 90 days, whereas the other groups exhibited similarly elevated risks of death or disability but without any increase in the odds of serious adverse cardiac or renal events.

Our findings add to the growing body of evidence supporting the importance of timely and effective SBP reduction to improve outcomes after acute ICH. Persistently high SBP, inadequate reduction, or overly aggressive lowering of SBP has been linked to poor outcomes after ICH. Large-scale pooled IPD analyses have demonstrated that earlier attainment and sustained maintenance of SBP within the 120–140 mm Hg range during the first 24 hours was significantly associated with a reduced risk of death or dependency.^1,11^ More recently, a post hoc analysis of the Rapid, Intensive, and Sustained BP lowering in Acute ICH study reinforced these findings, showing that achieving the SBP target within 1 hour of treatment initiation was linked to better clinical and functional outcomes.^12^ However, secondary analyses of the INTERACT2 and ATACH-II trials highlight potential harm from excessive and rapid SBP reductions, particularly in patients with very high baseline SBP. Specifically, reductions from >200 to <140 mm Hg may attenuate or reverse the benefits of BP-lowering treatment.^3^ In ATACH-II, patients with baseline SBP >220 mm Hg who underwent intensive SBP reduction to <140 mm Hg had higher rates of death or disability and more frequent renal and cardiovascular adverse events.^4^ Other analyses have also linked intensive SBP lowering in this subgroup to increased risk of neurologic deterioration.^13^ These findings underscore the need for a more individualized approach to BP management in acute ICH, balancing the potential benefits of early target attainment with the risks of overly aggressive lowering in certain high-risk patients.

We identified a subgroup of patients with severe hypertension who were at high risk of death or disability regardless of the SBP-lowering strategy used. This observation underscores the limitations of current guideline-based approaches, which emphasize gradual and sustained SBP reduction to minimize variability and prevent harm.^14^ Although such recommendations reflect a patient-centered philosophy, they are based on average treatment effects and do not account for the substantial interindividual heterogeneity in risk and response.^15,16^ Our findings highlight the need for a more individualized approach to acute BP management in ICH. Specifically, future work should focus on developing a therapeutic prediction tool that integrates key prognostic factors—including baseline SBP, age, hematoma volume, time to treatment, and comorbidities—to estimate individualized risks and benefits of SBP lowering. Such a tool could generate personalized targets and guide the intensity and timing of SBP reduction, thereby supporting more nuanced, data-driven clinical decisions. The stratified patterns of outcome observed in our study, particularly among high-risk subgroups, may inform the selection and weighting of predictor variables, as well as the thresholds at which different treatment strategies are recommended.

The strengths of these analyses include the large data set derived from participants recruited across a broad range of health care settings, with systematic evaluations of outcomes and high rates of adherence and follow-up. We acknowledge several limitations. First, statistical power may have been limited for some subgroup and interaction analyses, potentially obscuring modest associations. Second, the data-driven LCA approach is sample-specific and may not yield generalizable trajectory groups across different populations or settings. The derived classes depend on measurement timing, model specification, and population characteristics, limiting reproducibility. Third, because this was a secondary analysis within a trial cohort, residual confounding cannot be excluded and causal inferences should be made cautiously. External validation in independent cohorts is needed to confirm our findings and support broader applicability.

In summary, this study identified several distinct SBP trajectories in the first 24 hours after the onset of acute ICH, in which a subgroup of patients with severe hypertension had high odds of death or disability. These results highlight the need for tailored individualized SBP management in certain patients with acute ICH.

## Glossary

ATACH-II: second Antihypertensive Treatment of Acute Cerebral Hemorrhage
ICH: intracerebral hemorrhage
IPD: individual patient-level data
INTERACT: Intensive Blood Pressure Reduction in Acute Cerebral Hemorrhage Trial
IQR: interquartile range
LCA: latent class analysis
mRS: modified Rankin Scale
NIHSS: NIH Stroke Scale
OR: odds ratio
SBP: systolic blood pressure
